# Protection Efficacy of Multivalent Egg Yolk Immunoglobulin against *Eimeria tenella* Infection in Chickens

**Published:** 2013

**Authors:** JJ Xu, CZ Ren, SS Wang, DD Liu, LQ Cao, JP Tao

**Affiliations:** Ministry of Education Key Lab for Avian Preventive Medicine, Yangzhou University, Yangzhou, China

**Keywords:** IgY, Coccidiosis, *Eimeria tenella*, Passive immunization

## Abstract

**Background:**

To control avian coccidiosis with drug-independent strategy effectively and safely, multivalent hyperimmune egg yolk immunoglobulin (IgY) was prepared and its ability to protect against *Eimeria tenella* infection was evaluated.

**Methods:**

Hens were orally immunized with live oocysts of 5 species of *Eimeria* for six times, antibody titers in serum and yolk were monitored by indirect enzyme-linked immunosorbent assay. The specific IgY was isolated, purified and lyophilized. IgY powder was orally administrated as dietary supplement in newly hatched chicks at various dosages. Birds were orally challenged with 10000 sporulated oocysts of *E. tenella* at 10 days of age, weighed and killed at 8 days post challenge, and the protective effect was assessed.

**Results:**

The averge yeid of IgY was 9.2 mg/ml yolk, the antibody titer of IgY reached to 1:163840 per mg with the purity up to 98%. Chickens fed IgY resulted in reduced mortality, increased body weight gain (BWG), reduced oocyst shedding, reduced caecal lesion score and increased anti-coccidial index. In terms of BWG and caecal lesion, IgY significantly enhanced the resistance of bird at ≥ 0.05% of IgY in the diet when compared with the challenged control group (*P*<0.05). No significant difference was observed at dosage ≥ 0.5% and 1.0% when BWG and caecal lesion were compared with the sodium salinomycin control group, respectively (*P*>0.05).

**Conclusion:**

Supplementing newly hatched chicks with *Eimeria*-specific IgY represents a promising strategy to prevent avian coccidiosis.

## Introduction

Coccidiosis is a serious intestinal disease in chickens caused by seven species of *Eimeria* protozoan parasites which causes many losses in the poultry industry (1). Currently, coccidiosis is controled mainly with drugs. However, due to the increased drug resistance and concern of public health, alternative strategies such as live or subunit vaccines are requred and invented (2). Although vaccines played a role in coccidiosis control for a long time, their applications still remain very limited with consideration for the safety, costs and demands for high techniques of the farmers or veterinarians (3). Therefore, other drug-independent control strategies are urgently needed.

A few anticoccidial products have been tested as potential alternatives to drug or vaccine, these include herbal extracts (4), probiotics (5, 6), and antibodies (7–9). Among these, passive immunization with antibody is a highly attractive approach due to its high specificity. Generally, antibodies were obtained from chicken sera. However, this procedure is invasive, as chickens are usually sacrificed in order to obtain sufficient antibodies. Chicken egg yolk immunoglobin (IgY) is immunoglobin transferred from the serum of hens to the egg yolk, which offers passive immunity to newly hatched chickens. It has received special attention due to its non-invasive and cost-effective characteristics, and has been successfully used to control infectious diseases of chickens (10). Recently, a commerical hyperimmune egg yolk IgY powder named with Supracox^®^ against 3 species of *Eimeria* was demonstrated to provide certain protection against eperimental infections of *E.tenalla*, *E.maxima* and *E.acervulina* in chickens (11, 12). However, there is few details about the production, identification, and application in field of the Supracox^®^.

In China, the occurrence and distribution of *Eimeria* species vary in different areas, the predominant species have been reported to be *E. tenalla*, *E. maxima*, *E. praecox*, *E.acervulina* and *E.ecatrix* (13). In a previous study, we have identified an ideal *E.maxima* candidate for vaccine, which can provide chickens with good protection against experimental infections with up to 10 heterologous strains (14).

In the present study, we aimed to produce multivalent hyperimmune egg yolk IgY against the five predominant species of *Eimeria* in China. We also assessed its protective efficacy against experimental *E.tenalla* infection. Our data lay a foundation for alternative strategy to control coccidiosis in chickens.

## Materials and Methods

### Parasite, animal, antiserum

Wild strain of *E. tenalla*, *E. praecox*, *E.acervulina* and *E.necatrix* were isolated from Yangzhou, Jiangsu province, China, while *E. maxima* was isolated from Nantong in the same province. Sporulated oocysts were stored in 2.5% potassium dichromate solution at 4°C in the Key Laboratory for Avian Preventive Medicine, Yangzhou University. Parasites were propagated in chickens with conventional method every 2 months (15).

One-day-old yellow feathered broilers and Roman hens were provided by the Poultry Institute, Chinese Academy of Agricultural Science (Yangzhou, Jiangsu Province). Chikens were housed in *Eimeria*-free cages. Appropriate light, temperature, humidity, clean water and complete feed without anti-coccidia drug were provided according to the life stage and breed of chickens. Animal care and all experiments were reviewed and approved by the Animal Experiment Ethic Committee of Yangzhou University.

Chicken positive serum against five species of *Eimeria* was prepared as the follwing: broilers were orally immunized with mixed sporulated oocysts of *Eimeria* (2000 of *E. tenalla*; 1000 of *E. maxima*, *E. praecox, E. acervulina* and *E. necatrix*) at 10 days of age. The immunization was repeated every 2 weeks for five times. At one week after the last immunization, chickens were euthanized, serum was seperated and frozen in aliquots.

### Immunization of chickens

Roman hens were orally immunized with mixed sporulated oocysts of *Eimeria* (2000 of *E. tenalla*; 1000 of *E. maxima*, *E. praecox*, *E. acervulina and E. necatrix*) at 60 days of age. The hens were immunized for 6 times at intervals of 15 days. Eggs were collected daily for three months and stroed at 4°C for preparation of IgY. Eggs laid by non-immunized hens were collected and used as a negative control.

### Detection of antibody level in serum

At 0, 30, 60, 90, 120, 150, 180 and 210 days post the first immunization, 5 chickens were randomly selected and sera were collected to examine the specific antibody response against *Eimeria* with an enzyme-linked immunosorbent assay (ELISA). Briefly, 96-well microtiter plates were coated with soluble antigen of sporulated oocysts mixed with purified merozoites and gametophytes in carbonate buffer (0.05 M, pH 9.6). The plates were blocked with 10% fetal calf serum in PBS for 1 h at 37°C. Serial two-fold diluted serum sample was loaded to microtiter plate (100 µl/well) and incubated for 1.5 h at 37°C. After washing with PBS containing 0.05% Tween, peroxidase-conjugated rabbit-anti-chicken IgG (1:10000 dilution, Sigma, USA) was added (100 µl/well) and incubated for 1 h at 37°C. After washing, orthophenylene diamine (Sigma, USA) (0.4 mg/ml in 0.05 M phosphate citrate buffer, pH 5.0) was added (100 µl/well) and incubated for 15 min at 37°C to detect peroxidase activity. Finally, the reaction was stopped with sulfuric acid. Optical density at 490 nm (OD^490^) was measured with an automated microplate reader. There was PBS and serum from non-immunized hens in each plate, which served as a blank control and a negative control, respectively. Antibody titers were calculated as the maximal dilution multiple of the sample with value 2.1 times the negative control.

### Detection of antibody level in egg yolk

Eggs were collected from 5 hens at 0, 15, 30, 45, 60, 75, 90 and 105 days post-first laying. The yolk was first diluted with cold distilled water (acidified with 0.1 N HCl to pH 5.1), and incubated overnight at 4°C. The water soluble fraction (WSF) containing the IgY was collected by centrifugation at 10000×g at 4°C for 15 min. The specific antibody level in WSF was measured with ELISA according to the protocol mentioned in detection of antibody level in serum. There was PBS and supernatant of yolk from non-immunized hens in each plate, which served as a blank control and a negative control, respectively. Antibody titers were calculated as the maximal dilution multiple of the sample with value 2.1 times the negative control.

### Large-scale isolation and purification of IgY

IgY was isolated according to the water solution method described by Akita and Nakai with modifications (16). Briefly, egg separator was used to separate the yolk from the white. The yolk was thoroughly mixed with fresh and cold distilled water (V/V, 1:9). The mixture was adjusted to a pH value of 5.1 with HCl, and than incubated for 12 h at 4°C. The WSF containing the IgY was collected by centrifugation at 10000×*g* at 4°C for 15 min and then filtered through 0.22 µm membrane (Sangon Biotech Co., Ltd., Shanghai, China). Two-step salt precipitation was performed to purify the IgY in the supernatant in turn. First, ammmonium sulfate was added to 50% saturation, the mixture was stirred at 4°C for 1 h. Precipitate was collected by centrifugation at 10000×*g* at 4°C for 15 min and resuspened in deionized water. Secondly, ammmonium sulfate was added to 30% saturation, the mixture was stirred at 4°C for 1 h. Precipitate was again collected by centrifugation at 10000×*g* at 4°C for another 15 min and resuspened in PBS (0.01 M, pH 7.4). Finally, the precipitate was dialyzed against PBS and lyophilized.The lyophilized IgY powder was frozen at –20°C until further analysis and application.

### SDS-PAGE and antibody titer analysis of IgY

The quality and purity of IgY were identified by sodium dodecylsulfate polyacrylamide gel electrophoresis (SDS-PAGE). SDS-PAGE was conducted under reducing conditions with 5% stacking gel and 10% separation gel on an electrophoresis apparatus (BIO-RAD, USA). The protein bands were stained with Coomassie Brilliant Blue R250. The molecular weight of IgY was analyzed by Bio-Rad image analysis software, and the purity was analyzed by thin layer chromatography scanner (Shimadzu, Kyoto, Japan). IgY antibody activity was assessed with ELISA as described in detection of antibody level in serum.

### Experimental group and design

One-day-old yellow feathered broilers were divided into 9 groups, each with 20 chickens. Chickens in groups 1, 2, 3, 4, 5 and 6 were fed the standard diet well mixed with 0.01% IgY powder (w/w, IgY 0.01), 0.02% IgY powder (w/w, IgY 0.02), 0.05% IgY powder (w/w, IgY 0.05), 0.1% IgY powder (w/w, IgY 0.1), 0.5% IgY powder (w/w, IgY 0.5) and 1% IgY powder (w/w, IgY 1.0), respectively. Chickens in groups group 7 were fed the standard diet supplemented with 0.006% sodium salinomycin (w/w, SS 0.006). All supplements started from 1 day of age through the experiment. Chickens in group 8 were fed the standard diet and served as challenged control. Chickens in group 9 were fed the standard diet and served as unchallenged control. At 10 days of age, all birds were weighed, and those in groups 1 to 8 were orally challenged with 10000 sporulated oocysts of *E. tenella*. Clinical signs and mortality of each group were observed and documented daily. Feces of each group were collected separately at 5-8 days post infection (dpi). Chickens in all groups were weighed and euthanized on day 8 postchallenge. The detail for group and experimental design was shown in Table 1.

**Table 1:** Group and experimental design

Group	Number of chickens	Supplements	Concentrations (w/w)	Daysof supplement	Age of challenge (d)	Challenge dose of *E.tenalla* per chick
**1**	20	IgY powder	0.01%	18	10	1×10^4^
**2**	20	IgY powder	0.02%	18	10	1×10^4^
**3**	20	IgY powder	0.05%	18	10	1×10^4^
**4**	20	IgY powder	0.10%	18	10	1×10^4^
**5**	20	IgY powder	0.50%	18	10	1×10^4^
**6**	20	IgY powder	1.00%	18	10	1×10^4^
**7**	20	Sodium Salinomycin	0.006%	18	10	1×10^4^
**8**	20	None	None	0	10	1×10^4^
**9**	20	None	None	0	Unchallenged	unchallenged

### Evaluation of protective immunity

The efficacy of immunization was evaluated on the basis of survival rate, leison score, body weight gain(BWG), decrease ratio of oocyst and anti-coccidial index (ACI). The lesion score of caecum in each group was investigated according to Johnson and Reid by double-blind examination (17). BWG was determined at the beginning of the challenge, and at the end of the challenge. The relative weight gain was calculated as follows: (the average body weight gain from supplemented group/ the average body weight gain from unchallenged control group) × 100%. In each group, total oocysts in feces were calculated by a modified McMaster method (18), each sample was counted three times. The decrease ratio of oocyst was calculated as follows: (the average number of oocysts from challenged control group-the average number of oocysts from supplemented group)/ the average number of oocysts from challenged control group × 100%. ACI was calculated as follows: (relative weight gain + survival rate)-(lesion value + oocyst value) (17).

### Statistical analysis

Data were analyzed by SPSS software (SPSS 15.0 K for Windows, Chicago, IL). All data were expressed as mean ± S.D. values. Differences between the groups were determined using ANOVA. Duncan's multiple range test was used to analyze differences between the mean values, and differences were considered statistically significant at *P* < 0.05.

## Results

### Antibody titer in serum

**Fig. 1:**
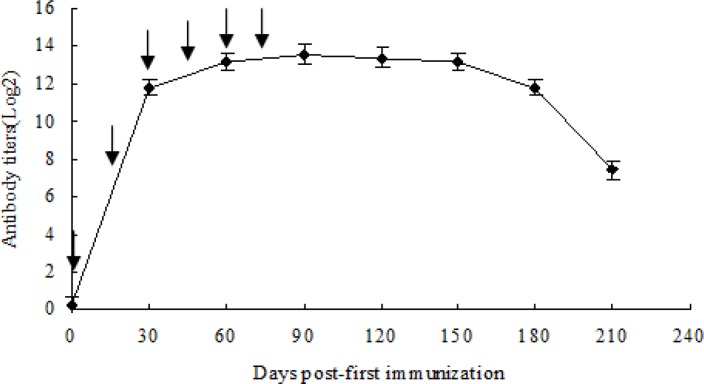
Antibody levels in serum of hens immunized with mixed sporulated oocysts post-first immunization (n = 5). Arrows indicate the six immunizations at intervals of 15 days. No antibody was detected in non-immunized hens.

The antibody titer against *Eimeria* in the serum of immunized hens increased over time, as revealed by ELISA (Fig.1). The antibody titer increased starting the first immunization, with the averge titer peaking at the day 5 after the sixth immunization, which reached to 1:13107.2. After 3 months post the the sixth immunization, the antibody titer rapidly declined.

### Antibody titer in yolk

**Fig. 2:**
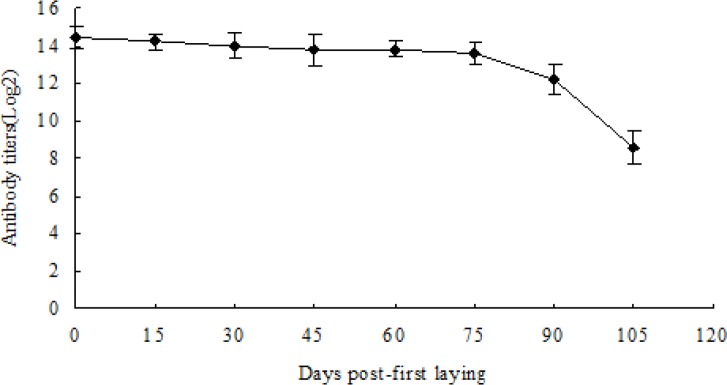
Antibody levels in water soluble fraction of yolks from eggs of hens immunized with mixed sporulated oocysts post-first laying (n = 5). No antibody was detected in non-immunized hens.

The antibody titer in water soluble fraction of yolk of hens post-first laying was monitored every 15 days with ELISA. As shown in Fig.2, the average titer of specific antibody reached a peak at the beging of the laying, and then declined very slowly. At 90 days post-first laying, the antibody titer rapidly declined. The antibody titer remained to be above 1:14175.6 for 75 days post-first laying.

### Purity and ELISA titer of IgY

**Fig. 3:**
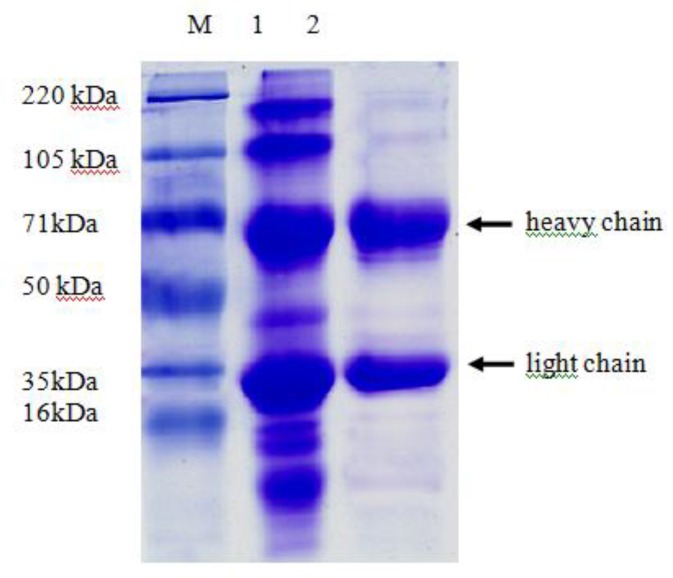
Sodium dodecylsulfate polyacrylamide gel electrophoresis (SDS-PAGE) pattern of purified egg yolk immunoglobulin (IgY). M, protein molecular weight marker; lane 1, water soluble fraction; lane 2, IgY purified by water dilution and ammonium preciptation. The arrows indicate the heavy chain (top arrow) and the light chain (bottom arrow) of IgY

**Fig. 4:**
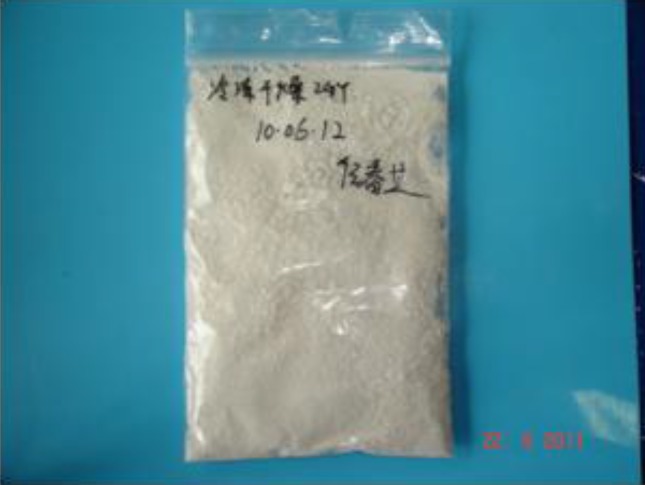
Sample of purified and lyophilized IgY powder

After water solution and two anmmonium sulfate precipitation, the purity of IgY was up to 98%, as revealed by SDS-PAGE and thin layer chromatography. There were two protein bands with molecular weight 68 kDa and 27 kDa, which represented the heavy chain and light chain of IgY, respectively (Fig.3). An image of the purified and lyophilized IgY powder was shown in Fig.4. The averge yield of IgY was 9.2 mg/ml egg yolk, and the antibody titer reached to 1:163 840 per mg of lyophilized powder.

### Protective effects of IgY against E. tenella challenge

The protective efficacy of IgY against *E. tenella* challenge in terms of survival rate, BWG, reduction in oocyst output, mean caecal lesion score and ACI is shown in Table 2.

**Table 2:** Protection efficacy of IgY against experimental infection of *E.tenella* in chickens

Group	survival rate (%)	Average body weight gain(g)	Relative body weight gain (%)	Mean leison scores	Oocyst output per chick (×10 ^6^)	Decrease ratio of oocyst(%)	Anticoccidial index
**IgY(0.01%)**	90	90.33±10.02^a^	49.91	3.50±0.17^ef^	9.025	27.43	84.91
**IgY(0.02%)**	95	97.00±10.58 ^ab^	53.59	3.29±0.24 ^ef^	7.789	37.37	95.69
**IgY(0.05%)**	100	114.67±11.37^bc^	63.35	3.13±0.26 ^e^	7.146	42.54	112.05
**IgY(0.10%)**	100	132.00±6.25^c^	72.93	2.69±0.37 ^d^	6.139	50.64	136.03
**IgY(0.50%)**	100	155.67±11.59^d^	86.01	2.21±0.17^c^	5.283	57.52	154.00
**IgY(1.00%)**	100	162.00±14.80^de^	89.50	1.91±0.13^bc^	4.074	67.46	160.40
**Sodium Salinomycin(0.006%)**	100	171.67±15.37^de^	94.85	1.78±0.17^b^	2.199	82.32	172.05
**Challenged control**	90	86.33±10.60 ^a^	47.70	3.59±0.13^f^	12.437	0	61.8
**Unchallenged control**	100	181.00±13.08^e^	100.00	0 ^a^	0	0	200

Note: data with different letters are significantly different at *P*<0.05.

Chickens provided with 0.01% IgY (IgY 0.01) had a 10% mortality, which was the same as the challenged control group. A 5% mortality was found in IgY 0.02 group. There was no mortality in IgY 0.05, IgY 0.1, IgY 0.5, IgY 1.0 and SS 0.006 groups.

There was no significant difference in BWG among the challenged control group, IgY 0.01 group and IgY 0.02 group (*P*>0.05). However, BWGs in IgY 0.05, IgY 0.10, IgY 0.50, IgY 1.0 and SS 0.006 groups were significantly higher than that in the challenged control group or IgY 0.01 group and IgY 0.02 group (*P*<0.05). Among these five groups, the highest BWG was observed in SS 0.006 group, then was the IgY 1.0 group and the IgY 0.5 group, but no significant difference was seen in these three groups (*P*>0.05).

A significant alleviation in cecal lesion was observed in the group fed sodium salinomycin compared with that of the challenged control group (*P*<0.05). Among the groups fed various concentration of IgY, the lesion score reduced gradually with the enhancement of concentration of IgY. Leison scores in IgY 0.05, IgY 0.10, IgY 0.50 and IgY 1.0 groups were significantly lower than that in the challenged control group (*P <*0.05). No significant difference was seen in lesion score between the SS 0.006 group and the IgY 1.0 group.

Compared with the challenged control, chickens fed IgY showed varied reduction in the number of oocysts in feces after infection with *E.tenella*. There was only 27.43% reduction in the number of oocysts in IgY 0.01 group, which was the lowest among the IgY groups. The highest reduction in the number of oocysts was seen in IgY 1.0 group, which was up to 67.46%. Other IgY groups had reductions of 37.37-57.52%. However, the decrease ratio of oocyst in SS 0.006 group was still higher than that in IgY 1.0 group, which was the highest among the groups fed IgY or sodium salinomycin.

Among all challenged groups, the ACI of the challenged control group was the least, and the ACI of sodium salinomycin group was the highest, which was up to172.05. Among the groups fed various concentration of IgY, the ACI increased gradually with the enhancement of concentration of IgY. The highest ACI was observed in IgY 1.0 group among IgY groups, even more than160.

## Discussion

IgY has shown remarkable resistance to extreme pH values, temperature and pepsin (10, 19). Oral adminsistration with specific IgY has shown to be effective against gastrointestinal pathogens such as *Escherichia coli*, rotavirus and *Helicobacter pylori* (20–22). However, little attention has been paid to the passive immunotherapy for intestinal parasitic diseases. Thus, the present study demonstrated the partial anticoccidial effect in chickens of IgY as a dietary supplement. The supplement resulted in reduced mortality, less oocyst shedding, lower caecal lesion score and increased ACI, as well as improvement of the growth performance of chickens infected with *E.tenella*. These data are consistent to the previous studies with commerical hyperimmune egg yolk IgY powder Supracox^®^ against three major *Eimeria* species (11, 12). Moreover, the IgY obtained in the present study contains polyclonal antibodies against five predominant *Eimeria* species in China, which was more than three species of Supracox^®^. Due to the differential distribution of *Eimeria* species in different regions, and the various antigenic variants of some species espically *E.maxima* (23), this multivalent characteristic may further expand the scope and improve the efficacy of IgY in the field, and cross protection against other species of chicken coccidia is clearly needed.

BWG and oocyst shedding are the main parameters of protective effect during experimental coccidiosis. However, one or two parameters did not truly reflect the protective effect, low correlations have often been reported among these parameters (5, 24–25). In the present study, BWG, oocyst output, lesion score and ACI were all used for the evalutation on protection of IgY, positive effects with all parameters were observed. This desirable correlation may be due to the direction of the multivalent IgY against many different life cycle stage of *Eimeria*, which is not achievable in many drugs (26).

In the present study, the dosage of IgY significantly affected the protection efficacy. Similar results were also seen in Supracox^®^ (11, 12). Compared with the challenged control group, IgY enhanced the resistance of bird against *E.tenella* at ≥ 0.05% of the diet in terms of BWG and mean caecal lesion (*P*<0.05). However, when compared to the sodium salinomycin group which showed the best protective effect among all challenged groups, no significant difference of BWG was only observed at dosage of 0.5% and 1.0% (*P*<0.05), while no significant difference was only observed at dosage of 1.0% in terms of mean caecal lesion (*P*<0.05), this may be associated with the antibody titer level of IgY. Further study should focus on the enhancement of antibody titer of IgY by optimization immunization of hens, which also reduced the cost and effective dose of IgY. Additionally, the infection dose of chickens in this study is considered to be much higher than that of broilers under field conditions (27), lower dose of IgY may be used in the field.

Compared to the challenged control, feeding the IgY powder at dosege of 1.0% prepared from un-immunized hens did not provide any protection (data not shown), this confirmed the protection effect of specific IgY. Cellular immunity and humoral immunity are both involved in the protective immunity in chickens against *Eimeria* infection^28^. Most studies have showed that cellular immunity is very critical, whereas the role of humoral immunity is minor (29). In contrast, partial studies also confirmed the ability of antibodies to block the invasion, development, and transmission of parasite (30). Passive and maternal immunity mediated by antibodies have been shown to be effective against against *Eimeria* infection (7, 8, 11, 12). Therefore, further study is needed to elucidate the immune mechanism mediated by the IgY.

The present study indicate that dietary administration of specific IgY could provide partial protection against *E. tenella* challenge in newly hatched chicks. This investigation is of interest for the development of new drug-independent control strategy against coccidiosis. However, its immune mechanism, optimal dose and administration procedure in the field remain to be explored. Further research is also required to enhance antibody titer of IgY and reduce the cost of production by optimizing the immunization regimes.

## Conclusion

Supplementing newly hatched chicks with *Eimeria*-specific IgY represents a promising strategy to prevent avian coccidiosis, and needs to be further evaluated in the field.
